# LAMP1 as a Target for PET Imaging in Adenocarcinoma Xenograft Models

**DOI:** 10.3390/ph18081122

**Published:** 2025-07-27

**Authors:** Bahar Ataeinia, Arvin Haj-Mirzaian, Lital Ben-Naim, Shadi A. Esfahani, Asier Marcos Vidal, Umar Mahmood, Pedram Heidari

**Affiliations:** 1 Division of Nuclear Medicine and Molecular Imaging, Department of Radiology, Massachusetts General Hospital, Harvard Medical School, Boston, MA 02114, USA; bahar.ataeinia@pennmedicine.upenn.edu (B.A.); ahajmirzaian@mgh.harvard.edu (A.H.-M.); lbennaim@mgh.harvard.edu (L.B.-N.); abdaresfahani.shadi@mgh.harvard.edu (S.A.E.); amarcosv@wi.mit.edu (A.M.V.); umahmood@mgh.harvard.edu (U.M.); 2 Center for Precision Imaging, Department of Radiology, Massachusetts General Hospital, Boston, MA 02114, USA

**Keywords:** LAMP1, Adenocarcinoma, Immuno-PET, breast cancer, colon cancer

## Abstract

**Background:** Lysosomal-associated membrane protein 1 (LAMP1), typically localized to the lysosomal membrane, is increasingly implicated as a marker of cancer aggressiveness and metastasis when expressed on the cell surface. This study aimed to develop a LAMP1-targeted antibody-based PET tracer and assess its efficacy in mouse models of human breast and colon adenocarcinoma. **Methods**: To determine the source of LAMP1 expression, we utilized human single-cell RNA sequencing and spatial transcriptomics, complemented by in-house flow cytometry on xenografted mouse models. Tissue microarrays of multiple epithelial cancers and normal tissue were stained for LAMP-1, and staining was quantified. An anti-LAMP1 monoclonal antibody was conjugated with desferrioxamine (DFO) and labeled with zirconium-89 (^89^Zr). Human triple-negative breast cancer (MDA-MB-231) and colon cancer (Caco-2) cell lines were implanted in nude mice. PET/CT imaging was conducted at 24, 72, and 168 h post-intravenous injection of ^89^Zr-DFO-anti-LAMP1 and ^89^Zr-DFO-IgG (negative control), followed by organ-specific biodistribution analyses at the final imaging time point. **Results**: Integrated single-cell and spatial RNA sequencing demonstrated that LAMP1 expression was localized to myeloid-derived suppressor cells (MDSCs) and cancer-associated fibroblasts (CAFs) in addition to the cancer cells. Tissue microarray showed significantly higher staining for LAMP-1 in tumor tissue compared to normal tissue (3986 ± 2635 vs. 1299 ± 1291, *p* < 0.001). Additionally, xenograft models showed a significantly higher contribution of cancer cells than the immune cells to cell surface LAMP1 expression. In vivo, PET imaging with ^89^Zr-DFO-anti-LAMP1 PET/CT revealed detectable tumor uptake as early as 24 h post-injection. The ^89^Zr-DFO-anti-LAMP1 tracer demonstrated significantly higher uptake than the control ^89^Zr-DFO-IgG in both models across all time points (MDA-MB-231 SUV_max_ at 168 h: 12.9 ± 5.7 vs. 4.4 ± 2.4, *p* = 0.003; Caco-2 SUV_max_ at 168 h: 8.53 ± 3.03 vs. 3.38 ± 1.25, *p* < 0.01). **Conclusions**: Imaging of cell surface LAMP-1 in breast and colon adenocarcinoma is feasible by immuno-PET. LAMP-1 imaging can be expanded to adenocarcinomas of other origins, such as prostate and pancreas.

## 1. Introduction

Cancer is a major cause of morbidity and mortality worldwide, with a predicted increase in its global burden over the following two decades. Adenocarcinomas of the breast, colon, prostate, and pancreas account for a significant proportion of cancer incidence and deaths, collectively representing approximately 40% of all new cancer diagnoses and resulting in over four million fatalities annually [1,2]. The indispensable role of imaging in oncological diagnosis and management has intensified the demand for innovative imaging agents capable of precise tumor localization and characterization, thereby optimizing patient selection for advanced therapeutic interventions [3]. Positron emission tomography (PET) has emerged as an essential tool in this context, providing insights into tumor biology and therapeutic responses at cellular and molecular levels [4]. The advent of immuno-PET, which synergistically combines the high sensitivity of PET with the specificity of monoclonal antibodies (mAbs), offers a promising avenue for enhancing precision in cancer imaging and targeted therapy [5,6].

Lysosomal-associated membrane proteins (LAMPs), particularly LAMP1, have attracted significant attention for their involvement in tumor progression [7,8]. LAMP1, initially characterized as a structural component essential for lysosomal integrity, is involved in processes such as lysosomal exocytosis and modulation of tumor cell–extracellular matrix (ECM) interactions [9,10]. Notably, upregulation of LAMP1 on the cell surface correlates with enhanced cancer cell adhesion, migration, and metastasis across multiple adenocarcinomas, including those of the colon, breast, and pancreas [11,12]. The role of LAMP1 in regulating the interaction between the cancer cells and TME components is increasingly recognized in different epithelial cancers, which is vital for metastasis [13]. In addition to tumor cells, LAMP1 expression in TME components such as cancer-associated fibroblasts (CAFs) could indicate the secretion of pro-oncogenic exosomes, which promote cancer cell invasion and metastasis [14]. This highlights the complex dynamics of the TME and underscores the potential of targeting LAMP1 as a theranostic strategy in cancer management. Although multiple immuno-PET tracers have been developed to image either tumor cells or components of the tumor microenvironment (TME), none provide a holistic strategy that integrates both. For example, CD8-targeted tracers enable visualization of cytotoxic T cell infiltration and have shown predictive value for immunotherapy response [15], while CD45-targeting agents offer high sensitivity for imaging all immune cells in the tumor [16]. Likewise, ^68^Ga-FAPI tracers selectively target cancer-associated fibroblasts, demonstrating remarkable uptake across 28 cancer types, particularly breast, esophageal, and lung cancers [17]. GRPR-targeted radiopharmaceuticals focus primarily on specific tumor cell subsets, showing promise in prostate and breast cancer imaging [18]. In contrast to these single-compartment approaches, LAMP-1 imaging simultaneously captures both tumor cells and key TME elements due to its high expression across these elements. This dual-targeting capability offers enhanced sensitivity and a more comprehensive characterization of the tumor, positioning LAMP-1 as an imaging biomarker with the potential to improve diagnostic sensitivity in adenocarcinomas.

In this study, we investigate the potential of LAMP1 as a novel imaging target in cancer diagnostics. We present evidence indicating that LAMP1 serves as a promising biomarker for the non-invasive detection of breast and colon adenocarcinomas.

## 2. Results

### 2.1. Single-Cell RNA Sequencing Reveals That LAMP1 Is Highly Expressed in Cancer Cells, MDSCs, and CAFs

**Figure 1:**
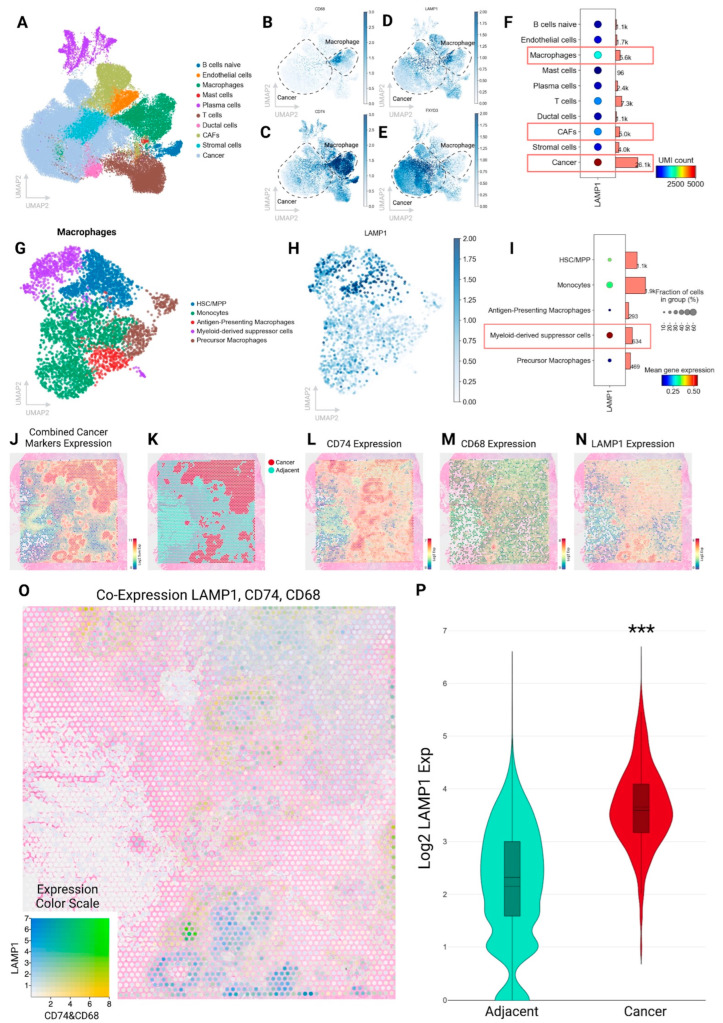
Integrated analysis of single-cell RNA sequencing and spatial transcriptomics reveals elevated LAMP1 expression in MDSCs and CAFs with enrichment in tumor regions compared to adjacent normal tissue. (**A**) K-means clustering analysis of single-cell RNA sequencing data highlights distinct cellular populations within the tumor microenvironment. Normalized log2 expression levels of CD68 (**B**), LAMP1 (**C**), CD74 (**D**), and FXYD3 (**E**) across all identified cell populations. (**F**) Quantitative analysis shows high expression of LAMP1 in cancer cells as well as macrophages and CAFs, which are prominent components of the tumor microenvironment. (**G**) Subtype clustering of macrophages identifies functional subsets, allowing for finer delineation of LAMP1 expression within specific macrophage populations. (**H**) Normalized log2 expression of LAMP1 within macrophage subtypes highlights differential expression patterns. (**I**) LAMP1 expression was significantly elevated in the MDSC subtype of macrophages. (**J**) Combined analysis of tumor marker expression within the spatial transcriptomics dataset reveals high expression of FXYD3, human epidermal growth factor receptor 2 (HER2), estrogen receptor (ER), MKI67, topoisomerase II alpha (TOP2A), and epithelial cell adhesion molecule (EPCAM). (**K**) Annotation of cancer cell regions in breast cancer tissue highlights tumor-specific regions. Spatial expression maps show CD74 (**L**), CD68 (**M**), and LAMP1 (**N**) in breast cancer tissue, with notable localization in cancer cells and tumor-associated immune cells. (**O**) Spatial co-expression analysis of CD74, CD68, and LAMP1 with a magnified view and (**P**) comparative analysis of LAMP1 expression between cancerous regions and adjacent normal tissue confirms a significant elevation of LAMP1 in tumor regions. ***: *p* < 0.001.

To map the LAMP1 expression in complex TME, we combined scRNA-seq and spatial RNA-seq analyses of breast tissue. Following data preprocessing, we clustered cells based on their gene expression profiles using K-means clustering, as illustrated in Figure 1A. Figure 1B–E depicts the normalized expression levels of CD68 (a macrophage marker), CD74 (a marker for myeloid-derived suppressor cells [MDSCs]), LAMP1, and FXYD3 (a ductal carcinoma marker) across all cells.

Notably, we observed that LAMP1 gene expression was high in cancer cells, followed by macrophages and cancer-associated fibroblasts (CAFs), as shown in Figure 1F. We performed a subgroup analysis to further investigate the source of LAMP1 expression among macrophages. We identified five distinct subpopulations of the macrophages (Figure 1G). Our analysis revealed that MDSCs exhibit significantly higher LAMP1 gene expression than other macrophage subpopulations (Figure 1H).

### 2.2. Spatially Resolved RNA Sequencing Reveals That the Anatomical Location of High LAMP1 Expression Tightly Matches the Tumoral Region

Given that cancer cells, MDSCs, and CAFs are the main sources of LAMP1 expression, we sought to determine whether LAMP1 expression correlates with the anatomical location of tumoral cells. Using spatial RNA sequencing annotated cancerous tissue using a combination of established breast cancer markers, including FXYD3, human epidermal growth factor receptor 2 (HER2), estrogen receptor (ER), MKI67, topoisomerase II alpha (TOP2A), and epithelial cell adhesion molecule (EPCAM) (Figure 1J–K), CD74 (MDSC marker; Figure 1L), CD68 (macrophage marker; Figure 1M), and LAMP1 (Figure 1N). Notably, co-expression analysis of LAMP1 with tumor markers CD74 and CD68 revealed that LAMP1 expression is mainly located within the tumoral area, potentially representing cancer cells, CAFs, and MDSCs (Figure 1O). Also, statistical spatial analysis confirmed that LAMP1 expression in the tumoral region was significantly higher than in normal adjacent tissue (Figure 1P). These findings suggest that LAMP1 could be a novel marker for visualizing both TME and cancer cells.

To explore LAMP1 as a novel epithelial cancer biomarker, we leveraged data from TCGA to compare LAMP1 expression levels in tumor versus normal samples across multiple cancer subtypes. Our analysis revealed that LAMP1 expression was significantly elevated in tumor samples for most cancer types (Supplementary Figure S1A). Additionally, we observed strong positive correlations between LAMP1 expression and key oncogenic markers, specifically ERK2 and EGFR (Supplementary Figure S1B,C). This suggests a role for LAMP1 in pathways associated with tumor progression. Functional analysis further indicated that silencing LAMP1 resulted in downregulating pathways critical to tumor development, including epithelial–mesenchymal transition (EMT), autophagy, and apoptosis (Supplementary Figure S1D–H). Collectively, our integrated single-cell, spatial RNA sequencing, and pan-cancer gene expression analyses provide compelling evidence that LAMP1 is being highly expressed within the tumor cells and TME elements such as MDSCs and CAFs.

### 2.3. Proteomic Assessment of LAMP1 Across Different Human Cancers

**Figure 2:**
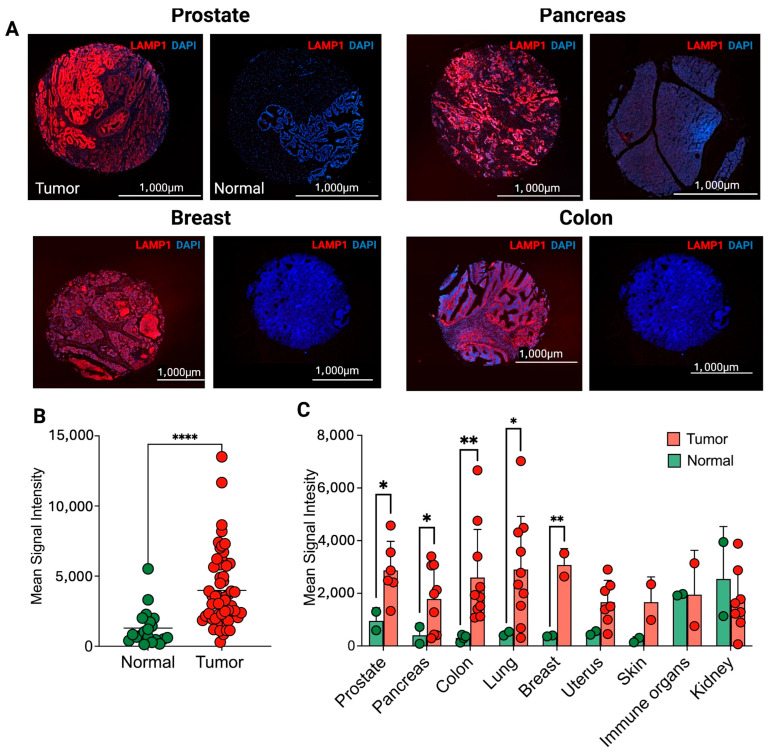
Assessment of LAMP1 expression in human pan-cancer samples and tumor-bearing murine model. (**A**) Representative immunofluorescence staining of LAMP1 (red channel) in major carcinomas and their respective normal tissue. Nuclei are stained with DAPI (blue channel). (**B**) The overall LAMP1 fluorescent signal intensity in tumor cores (red) versus normal cores (green). (**C**) Organ-based comparison of fluorescent signal intensity demonstrated significantly higher LAMP1 fluorescence in prostate, pancreas, colon, breast, and uterine endometrium carcinomas (red) compared to normal tissue (green). *, *p* < 0.05; **, *p* < 0.001; ****, *p* < 0.0001.

To evaluate LAMP1 abundance across various cancers, we quantified its expression using IF-based proteomics in matched human cancer and normal tissue samples, including prostate, breast, pancreas, colon, uterus, skin, kidney, and lymph nodes. Representative images for each cancer and normal tissue type are shown in Figure 2A. In the TMAs comprising diverse tumor types, cancerous TMA cores (n = 56) demonstrated significantly higher LAMP1 fluorescence intensity compared to normal tissue cores (n = 20, *p* < 0.001, Figure 2B). Specifically, as depicted in Figure 2C, fluorescent signal intensity was significantly elevated in cancer tissues relative to their normal counterparts for prostate (2872 ± 1105 vs. 948 ± 498, n = 6, *p* < 0.05), pancreas (1786 ± 909 vs. 948 ± 498, n = 9, *p* < 0.05), colon (2597 ± 1829 vs. 304 ± 128, n = 10, *p* < 0.01), uterus (1662 ± 830 vs. 494 ± 96, n = 7, *p* < 0.01), and breast (3077 ± 621 vs. 380 ± 25, n = 3, *p* < 0.001). Additionally, skin cancer exhibited a marginally significant increase in signal intensity compared to normal skin tissue (1666.25 ± 830 vs. 224.37 ± 95.81, n = 3, *p* = 0.08). However, no significant difference in LAMP1 intensity was observed between cancerous and normal tissues for renal carcinoma and lymph nodes (*p* > 0.05). To further characterize LAMP1 expression patterns, we employed cell-based flow cytometry analysis across five distinct adenocarcinoma cell lines: breast (MDA-MB-231), lung (KL205), colon (Caco-2), and pancreatic (SU86 and CAPAN2). Cells were incubated under two temperature conditions (4 °C and 37 °C) to assess LAMP1 internalization dynamics and trafficking under physiological versus non-physiological conditions. Flow cytometric analysis revealed temperature-dependent LAMP1 expression profiles. At physiological temperature (37° C), LAMP1-positive populations comprised 93.2%, 80.2%, 89.2%, 72.0%, and 83.8% for MDA-MB-231, KL205, Caco-2, SU86, and CAPAN2 cells, respectively. Notably, incubation at 4 °C resulted in substantially reduced LAMP1-positive populations: 54.3%, 52.9%, 53.5%, 52.1%, and 52.9% for the corresponding cell lines (Supplementary Figure S2). This temperature-dependent reduction suggests active endocytic trafficking of LAMP1 at physiological conditions. While all tested cell lines exhibited robust LAMP1 expression by flow cytometry, we selected MDA-MB-231 (breast adenocarcinoma) and Caco-2 (colon adenocarcinoma) as our primary models for subsequent experiments based on their superior expression profiles and experimental tractability.

### 2.4. Bioconjugation, Radiolabeling, and Binding Kinetics

The antibody was covalently immobilized onto a CM5 sensor chip (Cytiva). LAMP1 protein at a concentration series of 0–50 nM was injected over the channels at a 30 μL/min flow rate. Sensorgrams were corrected and fitted with Biacore. The results yielded a promising K_D_ of 1.78 ± 0.84 nM for human recombinant LAMP1 protein (Supplementary Figure S3A). We subsequently radiolabeled DFO-anti-LAMP1 with ^89^Zr^4+^ (Supplementary Figure S3B), achieving 95.9% purity and 85.4% labeling efficiency (Supplementary Figure S3C,D). In addition, cell binding assay saturation showed the K_D_ of 15.2 ± 0.01 nM (Supplementary Figure S4).

### 2.5. In Vivo Assessment of LAMP1 Expression in Tumor-Bearing Murine Model

**Figure 3:**
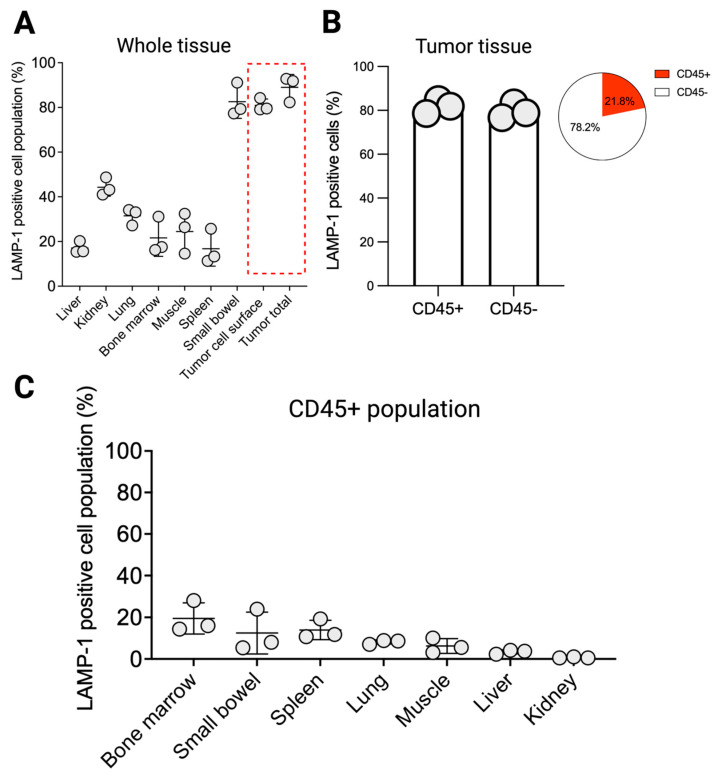
LAMP1 protein expression in tumor-bearing murine model. (**A**) LAMP1 positive cell population across major normal organs and tumor (cell surface and total) in tumor-bearing mouse model. (**B**) LAMP1 positive cell population proportion across CD45- and CD45+ cells in tumor tissue. (**C**) Total cell population of CD45 + LAMP1+ cell in major normal organs.

Using flow cytometry, we measured the cell surface expression of LAMP1 (without cell permeabilization) across various organs in a tumor-bearing murine model to evaluate the potential off-target sites for LAMP1-binding radiopharmaceuticals. We observed that cell surface LAMP1 was significantly lower in all examined organs and small intestine, compared to tumor (liver: 17 ± 3%, lung: 31 ± 3%, bone marrow: 22 ± 8%, kidney: 44 ± 4%, muscle: 24 ± 9%, spleen: 17 ± 8%, bone marrow: 89 ± 2% small intestine: 82 ± 7% vs. tumor: 81 ± 3%, *p* < 0.05, Figure 3A). Immune fluorescence imaging of normal tissue specimens showed that in normal organs such as the intestine and kidneys, LAMP1 is mainly expressed at the epithelial surface and is inaccessible for any radiopharmaceutical binding. Tumor cell membrane permeabilization showed only a modest 5–7% increase in LAMP1 expression, indicating that a substantial portion of LAMP1 in the tumor is membrane-bound (Figure 3A).

To evaluate the contribution of immune cells to tumor tissue LAMP1 expression, we gated for CD45+ LAMP1+ cell population. CD45+ immune cells comprised 21.8% of the total LAMP1+ cells within the tumor (Figure 3B). However, LAMP1 expression levels were similar between CD45+ and CD45− populations in the tumor (81.8% vs. 79.67%, respectively, Figure 3B). In other organs, such as the spleen, small intestine, and bone marrow, only up to 20% of the CD45+ population was LAMP1+. This indicates that tumor immune cells express LAMP1 at approximately four-fold higher levels than normal organs. These findings validate our single-cell and spatial RNA sequencing results and suggest that the radiopharmaceutical uptake in immune-rich organs is potentially low, resulting in a low background in these organs for in vivo imaging.

### 2.6. LAMP1 PET/CT Imaging Detects Tumors in a Tumor-Bearing Murine Model

**Figure 4:**
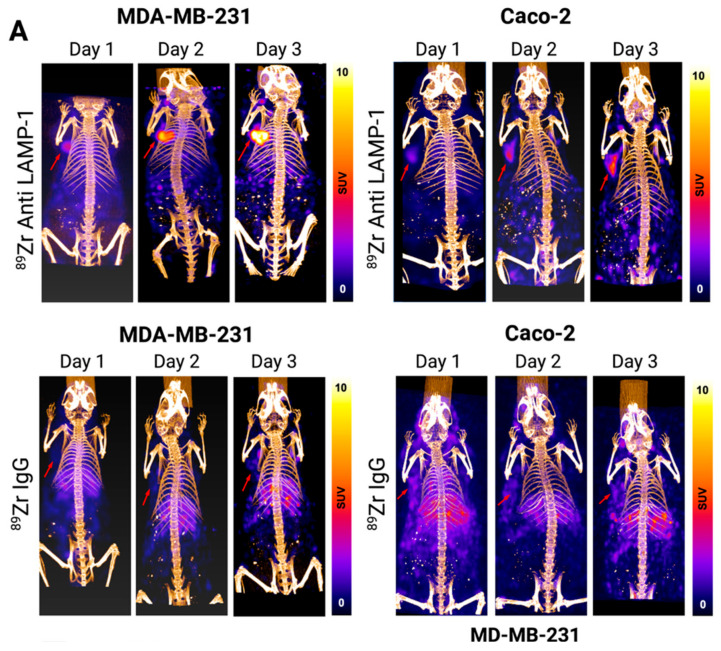

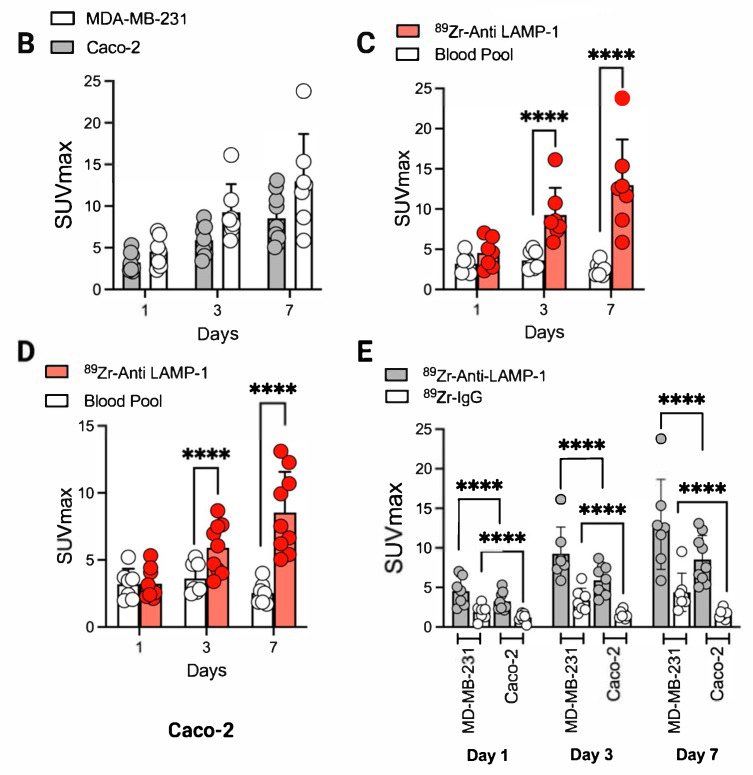
LAMP1 PET/CT imaging detects tumors in a tumor-bearing murine model at different time points. (**A**) Representative ^89^Zr-DFO-LAMP1 and ^89^Zr-DFO-IgG PET/CT images of MDA-MB-231 and Caco2 on day 1, day 3, and day 7. Red arrows point at the tumor. (**B**) Tumor SUV_max_ uptake of LAMP1 over the course of the experiment in MDA-MB-231 (white) and Caco2 (gray) reflected a sustained increase in tumor tracer localization. (**C**) Tumor SUV_max_ of ^89^Zr-DFO-LAMP-1 (red) in comparison to blood pool (white) in MDA-MB-231 (uptake in the heart region was used as a reference for the blood pool). (**D**) Tumor SUV_max_ of ^89^Zr-DFO-LAMP1 (red) in comparison to blood pool (white) in Caco-2 (uptake in the heart region was used as a reference for the blood pool). (**E**) Comparing the uptake of ^89^Zr-DFO-IgG (white) and ^89^Zr-DFO-LAMP1 (gray) in two MDA-MB-231 and Caco-2 during different time points in both models. ****, *p* < 0.0001.

For in vivo imaging, we generated tumor-bearing murine models using Caco-2 and MDA-MB-231 cells. Mice were administered ^89^Zr-DFO-anit-LAMP1 or ^89^Zr-DFO-IgG (control) as PET/CT tracers, and imaging was conducted on days 1, 3, and 7 post-injection. Figure 4A and Supplementary Figure S5 illustrate the tracer uptake dynamics over time for both groups. Tumor localization of the tracer was visible as early as 24 h post-injection, with minimal background accumulation in normal organs (Figure 3A,B and Figure S5). In the MDA-MB-231 group, the mean SUV_max_ was 4.5 ± 1.8, 9.2 ± 3.5, and 12.9 ± 5.7 on days 1, 3, and 7 post-injection, respectively, while in the Caco-2 group, SUV_max_ was 3.2 ± 1.1, 5.9 ± 1.8, and 8.5 ± 3.0 over the same time points (Figure 4B). In the MDA-MB231 model, comparing the tumor to blood pool showed significant increase on days 3 and 7 post-injection (*p* < 0.001, Figure 4C), and the tumor-to-blood ratio exhibited an increasing trend, rising from 1.4 ± 0.4 on day 1 to 2.8 ± 1.1 on day 3, and reaching 5.5 ± 3.8 on day 7 post-injection. Similarly, the Caco-2 model exhibited a significant increase at three and seven days post-injection (*p* < 0.001, Figure 4D), and the tumor-to-blood ratio showed an increasing trend from 1.1 ± 0.3 on day 1 to 2.9 ± 1.6 on day 3, and 4.3 ± 2.6 on day 7. Furthermore, ^89^Zr-DFO-anti-LAMP1 uptake was significantly higher than ^89^Zr-DFO-IgG uptake across all time points and models (Figure 4E, *p* < 0.001).

Biodistribution studies were conducted seven days post-injection in mice bearing breast and colon adenocarcinoma xenografts to evaluate the in vivo pharmacokinetics of ^89^Zr-DFO-anti-LAMP1 and the IgG control tracer. Specifically, in the breast cancer model, ^89^Zr-DFO-anti-LAMP1 uptake was substantially higher than that of the IgG control (%ID/g 21.79 ± 5.77 vs. 7.69 ± 3.90, *p* < 0.001, Supplementary Figure S6A), and in the colon adenocarcinoma model, the tracer exhibited significantly higher retention in tumor tissue than in normal colon tissue (%ID/g 11.75 ± 4.69 vs. 1.07 ± 0.37, *p* < 0.0001, Supplementary Figure S6B). As anticipated, the uptake in the liver and spleen is higher than in other normal organs, which is expected from an antibody-based imaging tracer. These results demonstrate the potential of ^89^Zr-DFO-anti-LAMP1 as a tumor PET imaging probe in vivo, with specific uptake in the tumor and limited off-target accumulation.

## 3. Discussion

This study presents compelling evidence that LAMP1 is a promising imaging biomarker for the non-invasive detection of epithelial cancers such as breast and colon adenocarcinomas. Utilizing an anti-LAMP1 mAb PET tracer, we observed significant tumor uptake as early as 24 h post-injection, with tracer accumulation and retention persisting for up to 7 days. This rapid and increasing uptake underscores the potential of LAMP1-based radiopharmaceuticals for imaging and therapeutic applications across a spectrum of human carcinomas, such as colon, breast, and prostate cancers [19]. The high expression of LAMP1 in various human carcinomas suggests its utility as a versatile target for diagnostic and therapeutic oncology strategies [20,21].

Despite the established association of LAMP1 with tumor biology, the precise mechanisms by which LAMP1 influences tumor progression, metastasis, and treatment resistance remain inadequately understood [22,23]. Notably, alterations in the glycosylation patterns of LAMP1 have been implicated in enhancing cancer cell adhesion to the endothelium and facilitating metastasis [24,25]. It has been highlighted that MDSCs are significantly influenced by the TME, characterized by the presence of various cytokines and chemokines that promote their accumulation and activation [26,27,28]. For instance, interleukin-6 (IL-6) and other inflammatory mediators in the TME enhance the immunosuppressive functions of MDSCs, leading to increased tumor growth and metastasis [25,28,29]. This suggests that LAMP1, which interacts with CAFs, might be upregulated in response to such inflammatory signals, while it contributes to the recruitment and enhancement of MDSCs [28]. Moreover, the expression of LAMP1 on tumor cells facilitates their interaction with MDSCs, potentially enhancing the immunosuppressive environment that tumors exploit to evade immune detection [30]. This interaction may be mediated through various signaling pathways activated by the TME, including those involving transforming growth factor-beta (TGF-β) and other cytokines that modulate MDSC activity [31]. Therefore, upregulation of LAMP1 in these contexts may serve as a marker for increased MDSC activity and a more aggressive tumor phenotype, suggesting that targeting LAMP-1 could be a potential therapeutic strategy to disrupt the immunosuppressive network established by MDSCs. Interestingly, in this study, by leveraging integrated single-cell and spatially resolved RNA sequencing, we demonstrated that in the TME, besides the cancer cells, MDSCs and CAFs are the primary sources of LAMP1 expression. Additionally, through pan-cancer bioinformatic analysis, we observed a high abundance of LAMP1 across various cancer types. Collectively, these findings strongly suggest that a LAMP1-targeted probe could serve as a novel and promising tracer for imaging and potentially the treatment of epithelial cancers. Future clinical development should also focus on optimizing the theranostic potential of LAMP1 targeting through the development of therapeutic radioisotope conjugates that can exploit the same targeting mechanism while managing the challenge of off-target organ exposure. The clinical translation of ^89^Zr-DFO-anti-LAMP1 will necessitate comprehensive dosimetry and manufacturing in compliance with Good Manufacturing Practice (GMP) standards. The hepatic and splenic uptake observed in preclinical studies indicates that these organs may serve as dose-limiting factors, thereby requiring voxel-level dosimetry and human dose modeling based on biodistribution data to determine safe activity thresholds. GMP production will involve sourcing GMP-grade ^89^Zr or establishing cyclotron-based ^89^Zr production workflows with automated target processing, along with radionuclidic and radiochemical purity assessments. Additionally, this process will require GMP-adapted DFO-antibody conjugation with strict release criteria concerning immunoreactivity, stability verification, sterility, and apyrogenicity. The translation process will proceed following IND-enabling toxicology and dosimetry studies, as well as early-phase clinical trials in patients with adenocarcinoma exhibiting unmet imaging needs.

In this study, for the first time, we developed a novel ^89^Zr-DFO-mAb PET tracer targeting LAMP-1 and demonstrated its potential as an imaging biomarker for the non-invasive detection of breast and colon adenocarcinomas. Our results show that tumors were visible on PET/CT as early as 24 h post-injection, with significant tracer accumulation and retention up to 7 days. These findings were confirmed through ex vivo biodistribution studies. We also demonstrated the potential applicability of LAMP1-based PET for a variety of carcinomas by performing LAMP1 immunofluorescence staining on human colon, breast, and prostate carcinoma samples, as well as genomic analysis.

One of the primary limitations of our study was the absence of a negative control xenograft model, as most epithelial cancer cell lines express LAMP1, making it challenging to identify a truly LAMP1-negative cell line for comparison. To address specificity concerns, we employed rabbit IgG as a control probe to demonstrate the specificity of our LAMP1-targeting probe compared to non-specific antibody binding. However, we acknowledge that our study lacks in vivo blocking experiments using excess unlabeled anti-LAMP1 antibody, which represents a significant limitation in definitively establishing receptor-mediated specificity. While IgG controls are necessary to assess non-specific uptake, blocking studies with cold antibody would provide more robust evidence for specific receptor-mediated uptake mechanisms. Additionally, we observed lower tumor uptake in Caco-2 xenografts compared to MDA-MB-231 models, which may reflect differences in LAMP1 expression levels, tumor vascularization, or antibody penetration between these cancer types. This variability in uptake across different cancer models suggests that the clinical translation of LAMP1-targeted imaging may require optimization for specific cancer subtypes. Future studies incorporating comprehensive blocking experiments and expanded cancer models would strengthen the evidence for LAMP1-specific targeting and broaden the potential clinical applications of this imaging approach. Finally, although at the end of the study we evaluated the biodistribution and performed multiple time point PET scans, having multiple time point biodistribution studies might be helpful to understand the pharmacokinetics of the radioligand.

## 4. Materials and Methods

### 4.1. Integrated Single-Cell RNA Sequencing and Spatial Transcriptomic Analysis

We employed an integrated approach combining single-cell RNA sequencing (scRNA-seq) and spatial transcriptomics to investigate breast cancer biology (GSE243280). First, raw FASTQ files were downloaded from the NCBI server, and Cell Ranger software (10× Genomics, Cell Ranger v7.1.0, Pleasanton, CA, USA) was used for preliminary data processing of both scRNA-Seq and spatial transcriptomics datasets [32]. We analyzed the output using Scanpy (version 2.6.1) and Loupe Browser (10× Genomics Loupe Browser v8.0.0, Pleasanton, CA, USA) for comprehensive cell type identification and spatial visualization [33]. For broader, pan-cancer RNA expression analyses, we utilized online resources such as TNMplot.com [34], Gepia2 [35], and TIMER.2 [36] to examine RNA expression across various cancer types. Additionally, we referenced the GSM2059432 dataset to assess the impact of LAMP1 knockdown on the gene expression profile in cancer [37]. We performed KEGG pathway analysis and gene set enrichment analysis tool (GSEA, version 4.3.3) by using Gene Ontology Molecular Functions (GO:MF), HALLMARK pathways, and oncogenic signature gene data sets, to highlight pathways and functions potentially influenced by knocking down LAMP1 [38].

### 4.2. Immunofluorescence Staining of Human Tissue Samples

All human tissue microarrays (TMAs) of the colon (Tissue Array Cat# CO1507), breast (Tissue Array Cat# BR1191), and prostate (Tissue Array Cat# PR781) were purchased from TissueArray.Com LLC (Derwood, MD, USA). Samples were deparaffinized and rehydrated, and heat-based antigen retrieval was performed [39]. Samples were incubated with clone D2D11 rabbit anti-human LAMP-1 (Cell Signaling, Danvers, MA, USA, Cat# 9091S) overnight at 4 °C. The following day, stained slides were washed and incubated with alpaca AF647-conjugated anti-rabbit IgG secondary antibody (Jackson Immunoresearch, Grove, PA, USA, Cat# 611-605-215, RRID# AB_2721876) and DAPI (Abcam, Cambridge, UK, Cat# ab104139) for nuclei staining. The slides were scanned using the Aperio ScanScope^®^ System (Software version 12.4.6, Aperio Technologies, Vista, CA, USA). The TMA images were analyzed with Qupath (v0.5.1) [40]. Tissue segmentation was performed based on the brightfield images registered to the DAPI and far-red fluorescence channels (Cy5) to label the regions of the images containing tissue. The normalized intensity of the Cy5 was calculated for each region of interest (ROI).

### 4.3. Cancer Cell Lines and Cell Culture

All human and murine cancer cell lines were purchased from the American Type Culture Collection (ATCC, Manassas, VA, USA), except for MC38 cells (Kerafast, Boston, MA, USA, Cat# ENH204-FP) and HY15549 (a gift from Dr. Kenneth Tanabe, Massachusetts General Hospital, Boston, MA, USA). Human cancer cell lines include MDA-MB-231 (breast, ATCC Cat# HTB-26, RRID# CVCL_IN16), Caco2 (colon, ATCC Cat# HTB-37, RRID# CVCL_0025), SU86.86, and CAPAN2 (pancreas), and KL205 (lung, ATCC Cat# CRL-1453, RRID# CVCL_3533). Cells were grown using DMEM (ThermoFisher, Waltham, MA, USA, Cat# 12491023) or RPMI (ATCC) medium according to the vendor’s recommendation, supplemented with 10% fetal bovine serum (ThermoFisher, Cat# A5669701) and 1% penicillin/streptomycin (Thermo Fisher, Car# 15140122) at 37 °C and 5% CO_2_ incubator.

### 4.4. Xenograft and Allograft Models

All animals were housed under standard specific pathogen-free (SPF) conditions in individually ventilated cages with controlled temperature (20–24 °C), humidity (40–60%), and a 12-h light/dark cycle. Animals had ad libitum access to autoclaved food and water and were monitored daily for general health and signs of distress. The Institutional Animal Care and Use Committee (IACUC) approved all experimental procedures and animal studies. Mouse models of human breast and colon adenocarcinoma were generated with subcutaneous injection of 2 × 10^6^ cells in a 1:1 (*v*:*v*) ratio in Matrigel (Corning Inc., Corning, NY, USA, Cat# CB354248) into the left shoulder of athymic nude mice (Charles River Laboratories, Wilmington, MA, USA). Female mice were used for the MDA-MB-231 breast, and males were used for the Caco-2 colon cancer model. Once the tumor size reached approximately 150 mm^3^, mice were randomly assigned to the experimental ^89^Zr-DFO-LAMP1 or control ^89^Zr-DFO-IgG probe groups (n = 7 per group).

To evaluate the contribution of immune cells in organs to the expression of LAMP-1 in tumors and normal organs, we used the syngeneic allograft MC38 model in immunocompetent C57BL/6 (Jackson Laboratories, Bar Harbor, ME, USA) mice. MC38 tumors were implanted subcutaneously in the left shoulder of male mice, as described. Once the tumors reached 150–250 mm^3^, mice were euthanized. Tumors and major organs were harvested and processed for flow cytometry (n = 3).

### 4.5. Flow Cytometry

Cells were dissociated using a non-enzymatic cell dissociation solution (ATCC, Cat#30-2103). Human cell lines were stained with AlexaFluor 647 (AF 647)-conjugated clone D2D11 LAMP-1 antibody (Cell Signaling, CaT# 73589S) and murine cell lines with APC-conjugated anti-mouse LAMP-1 antibody (BioLegend, San Diego, CA, USA, Cat# 121613, RRID# AB_2234505). Next, cells were stained with Zombie Aqua viability dye (Biolegend, Cat# 423101). Cells were fixed in paraformaldehyde 2% and run on the LSRFortessa X20 flow cytometer (BD Biosciences, San Jose, CA, USA). To examine the in vivo expression of LAMP-1 in MC38 tumor-bearing mice, the entire tumor, liver, lung, spleen, kidneys, small bowel, bone marrow, and thigh muscle were harvested, and cells were mechanically dissociated. Bone marrow was further processed in RBC lysis buffer (Biolegend, Cat# 420301). Cells were stained with APC-conjugated anti-mouse LAMP-1 antibody (BioLegend Cat# 121613, RRID# AB_2234505), using the above protocol. All flow cytometry data were analyzed using FlowJo software (version 10.1).

### 4.6. Bioconjugation, Radiolabeling of Antibodies, and Binding Kinetics Analysis

All the buffers were treated with Chelex-100^®^ resin (Sigma Aldrich, St. Louis, MO, USA, Cat# C7901-100G) to ensure metal-free conditions. Rabbit anti-human LAMP1 mAb (Novus Biological, Centennial, CO, USA, Cat# NBP2-89844, RRID# AB_3442448) was conjugated to p-SCN-Bn-deferoxamine (DFO) chelator (Macrocyclics, Plano, TX, USA, CAT# B-705) before radiolabeling with ^89^Zr according to the standard methods [41]. Briefly, a solution of 2 mg DFO-anti-LAMP1 in 0.5 M HEPES (pH 7.5) was reacted with 6.0 mCi of pH-adjusted ^89^Zr (from 1 M oxalic acid stock, neutralized with 1 M Na_2_CO_3_ to pH 6.8–7.5), incubated for 60 min at room temperature with agitation, and radiolabeling yield assessed via radio-TLC using 50 mM DTPA (pH 5.5) as the mobile phase. The reaction was quenched with DTPA, purified by size exclusion column using gentisic acid-containing buffer to protect against radiolysis, and the final product yield, purity, and specific activity were calculated.

The rabbit IgG control antibody (Millipore Sigma, St. Louis, MO, USA, CAT# I5006, RRID# AB_1163659) was conjugated and labeled using the same protocol. The antibodies were conjugated in a reaction with a 4-fold molar excess of p-SCN-Bn-DFO. The binding kinetics of DFO-anti-LAMP1 mAb with human recombinant LAMP1 protein (R&D Systems, Minneapolis, MN, USA, Cat# 4800-LM-050) were analyzed by surface plasmon resonance (SPR) using a BIACORE T200 instrument (Cytiva, Marlborough, MA, USA) [42]. The DFO substitution level of the mAb (chelators/molecule) was measured by labeling a 10 μL aliquot of the unpurified conjugate with ^89^Zr and then determining the proportion of ^89^Zr-DFO-anti-LAMP1 vs. free ^89^Zr-DFO by ITLC-SG and multiplying this fraction by the molar ratio used in the reaction as previously described [43]. Using this method, we calculated approximately 1.5 DFO chelator molecules per antibody molecule.

### 4.7. PET/CT Imaging and In Vivo Biodistribution Analysis

PET imaging was performed 24 (day 1), 72 (day 3), and 168 h (day 7) after tail vein injection of approximately 7.5 MBq of either ^89^Zr-DFO-LAMP-1 or ^89^Zr-DFO-IgG. Mice were anesthetized by inhalation of 2% isoflurane, and PET/CT images were acquired on a SuperArgus PET/CT scanner (Sedecal, Madrid, Spain). PET acquisition was performed in whole body mode with two-bed positions for 20 min per bed, followed by CT acquisition. Images were reconstructed using a 2D-OSEM algorithm (2 iterations and 20 subsets) and corrected for scatter and randoms. PET/CT images were processed using VivoQuant software (InviCRO, Burlington, MA, USA, version 5.2). A 3D ROI was manually drawn and segmented around the tumor region and heart (as blood pool) using CT images for anatomic reference. Mean and max standardized uptake values (SUVs) for each ROI were measured.

Following the last PET/CT scan time point, mice from experimental and control groups were euthanized. Blood samples and the major organs were collected and weighed, and their decay-corrected radioactive contents were measured using a Wizard-2 γ counter (PerkinElmer, Waltham, MA, USA). Results of the biodistribution studies are presented as percent injected dose per gram of tissue (%ID/g).

### 4.8. Statistical Analysis

Statistical analysis was performed using GraphPad Prism (version 10). Quantitative data are presented as the mean ± standard deviation. Direct comparisons between groups were made using a two-sided Student’s *t*-test. A *p*-value less than 0.05 was considered statistically significant. The fluorescence signal intensity in tissue cores was compared using the non-parametric Mann–Whitney U test after a normality Shapiro test. All analyses were conducted in a blinded manner with the analyst unaware of group assignments.

## 5. Conclusions

In conclusion, our study presents compelling evidence that LAMP1 holds significant promise as a biomarker for the non-invasive imaging of a diverse carcinomas, particularly breast and colon cancers. Given the strong relation of LAMP1 with tumor growth, invasion, aggressiveness, as well as an immunosuppressive TME, LAMP1 PET imaging should be further investigated as a prognostic biomarker. In addition, the high abundance of LAMP1 expression in various epithelial cancers and relatively low expression or inaccessibility in normal organs suggests that it could be a prime target for radiopharmaceutical-based therapies. Further investigations are warranted to assess LAMP1-based radiopharmaceuticals’ performance in the theranostic approach to these malignancies, especially for cancers with high mortality and limited therapeutic options.

## Data Availability

All processed data presented in the study are contained within manuscript and Supplemental Materials.

## Supplementary Materials

The following supporting information can be downloaded at: https://www.mdpi.com/article/10.3390/ph18081122/s1, Figure S1. (A) Pan-cancer LAMP-1 RNA expression in tumors (blue) and normal tissue (red). (B) Positive significant correlation between LAMP-1 and ERK-2 expression. (C) Positive significant correlation between LAMP-1 and EGFR expression. (D) LAMP-1 expression was higher in pan-cancer doxorubicin non-responders compared to responders. (E) The top significant down-regulated signatures in Hallmark gene sets for RNASeq analysis. The blue highlighted down-regulated signatures. GSEA analysis on signatures of (F) Epithelial mesenchymal transition. (G) Apoptosis and (H) Autophagy; Figure S2. Flow cytometry analysis of LAMP1 expression in cancer cell lines under different temperature conditions; Figure S3. (A) Binding affinity by SPR. Representative SPR sensorgrams show the response over time (resonance units, RU) during the association and disassociation binding phases of immobilized anti-LAMP-1 IgG Ab to recombinant human LAMP-1 (1:1 fit is shown as black curves). The anti-LAMP-1 IgG binds LAMP-1 at an affinity of KD = 1.78 ± 0.84 nM. (C) Representative radio-TLC chromatograms on ^89^Zr-DFO-LAMP-1 of crude radioimmunoconjugate. (B) Schematic representation of DFO conjugation and ^89^Zr labeling. (D) Binding affinity after purification (E), which resulted in filtering the unbound ^89^Zr; Figure S4. Dose–response curve for specific ligand binding in the saturation cell-binding assay; Figure S5. Cell blocking assay shows that blocking with 10× molar excess cold anti-LAMP-1 mAb significantly reduces the cell uptake in of DUBCA cells transduced with human LAMP-1 compared to non-blocked cells indicating specificity for the target; Figure S6. Biodistribution of ^89^Zr-DFO-LAMP1 (red) and ^89^Zr-DFO-IgG (white) in tumor and major organs in (A) MDA-MB-231 and (B) Caco2 demonstrated high tumor uptake and low normal organ retention of ^89^Zr-DFO-LAMP1.

## References

[1] Bray F., Ferlay J., Soerjomataram I., Siegel R.L., Torre L.A., Jemal A. (2018). Global cancer statistics 2018: GLOBOCAN estimates of incidence and mortality worldwide for 36 cancers in 185 countries. CA Cancer J. Clin..

[2] Siegel R.L., Miller K.D., Fuchs H.E., Jemal A. (2021). Cancer statistics, 2021. CA Cancer J. Clin..

[3] Chin C.N., Subhawong T., Grosso J., Wortman J.R., McIntosh L.J., Tai R., Braschi-Amirfarzan M., Castillo P., Alessandrino F. (2022). Teaching cancer imaging in the era of precision medicine: Looking at the big picture. Eur. J. Radiol. Open.

[4] Schwenck J., Sonanini D., Cotton J.M., Rammensee H.-G., la Fougère C., Zender L., Pichler B.J. (2023). Advances in PET imaging of cancer. Nat. Rev. Cancer.

[5] Boerman O.C., Oyen W.J. (2011). Immuno-PET of cancer: A revival of antibody imaging. J. Nucl. Med..

[6] Alauddin M.M., Khawli L.A. (2021). Advances in Immuno-PET for the Detection of Cancer and Assessment of Response to Therapy. Curr. Med. Chem..

[7] Tan K.-P., Ho M.-Y., Cho H.-C., Yu J., Hung J.-T., Yu A.L.-T. (2016). Fucosylation of LAMP-1 and LAMP-2 by FUT1 correlates with lysosomal positioning and autophagic flux of breast cancer cells. Cell Death Dis..

[8] Xu Y., Cao X., Zhang S., Zhang Y., Shen Z. (2017). High expression of LAMP1 as a prognostic marker in patients with epithelial ovarian cancer. Int. J. Clin. Exp. Pathol..

[9] Alessandrini F., Pezzè L., Ciribilli Y. (2017). LAMPs: Shedding light on cancer biology. Seminars in Oncology.

[10] Jensen S.S., Aaberg-Jessen C., Christensen K.G., Kristensen B. (2013). Expression of the lysosomal-associated membrane protein-1 (LAMP-1) in astrocytomas. Int. J. Clin. Exp. Pathol..

[11] Wang Q., Yao J., Jin Q., Wang X., Zhu H., Huang F., Wang W., Qiang J., Ni Q. (2017). LAMP1 expression is associated with poor prognosis in breast cancer. Oncol. Lett..

[12] Chen H., Li L., Hu J., Zhao Z., Ji L., Cheng C., Zhang G., Zhang T., Li Y., Chen H. (2019). UBL4A inhibits autophagy-mediated proliferation and metastasis of pancreatic ductal adenocarcinoma via targeting LAMP1. J. Exp. Clin. Cancer Res..

[13] Agarwal A.K., Srinivasan N., Godbole R., More S.K., Budnar S., Gude R.P., Kalraiya R.D. (2015). Role of tumor cell surface lysosome-associated membrane protein-1 (LAMP1) and its associated carbohydrates in lung metastasis. J. Cancer Res. Clin. Oncol..

[14] Xi L., Peng M., Liu S., Liu Y., Wan X., Hou Y., Qin Y., Yang L., Chen S., Zeng H. (2021). Hypoxia-stimulated ATM activation regulates autophagy-associated exosome release from cancer-associated fibroblasts to promote cancer cell invasion. J. Extracell. Vesicles.

[15] Kist de Ruijter L., van de Donk P.P., Hooiveld-Noeken J.S., Giesen D., Elias S.G., Lub-de Hooge M.N., Oosting S.F., Jalving M., Timens W., Brouwers A.H. (2022). Whole-body CD8+ T cell visualization before and during cancer immunotherapy: A phase 1/2 trial. Nat. Med..

[16] Salehi Farid A., Rowley J.E., Allen H.H., Kruger I.G., Tavakolpour S., Neeley K., Cong M., Shahbazian H., Dorafshani N., Berrada A. (2025). CD45-PET is a robust, non-invasive tool for imaging inflammation. Nature.

[17] Ma Y., Gao F. (2024). Advances of radiolabeled GRPR ligands for PET/CT imaging of cancers. Cancer Imaging.

[18] Kratochwil C., Flechsig P., Lindner T., Abderrahim L., Altmann A., Mier W., Adeberg S., Rathke H., Röhrich M., Winter H. (2019). 68Ga-FAPI PET/CT: Tracer uptake in 28 different kinds of cancer. J. Nucl. Med..

[19] Parks A., Charest-Morin X., Boivin-Welch M., Bouthillier J., Marceau F. (2015). Autophagic flux inhibition and lysosomogenesis ensuing cellular capture and retention of the cationic drug quinacrine in murine models. PeerJ.

[20] Zhang Y., Jiang X., Deng Q., Gao Z., Tang X., Fu R., Hu J., Li Y., Li L., Gao N. (2019). Downregulation of MYO1C mediated by cepharanthine inhibits autophagosome-lysosome fusion through blockade of the F-actin network. J. Exp. Clin. Cancer Res..

[21] Duangkumpha K., Stoll T., Phetcharaburanin J., Yongvanit P., Thanan R., Techasen A., Namwat N., Khuntikeo N., Chamadol N., Roytrakul S. (2019). Urine proteomics study reveals potential biomarkers for the differential diagnosis of cholangiocarcinoma and periductal fibrosis. PLoS ONE.

[22] Martins I., Wang Y., Michaud M., Ma Y., Sukkurwala A., Shen S., Kepp O., Métivier D., Galluzzi L., Perfettini J. (2014). Molecular mechanisms of ATP secretion during immunogenic cell death. Cell Death Differ..

[23] Ng Y.-Z., Pourreyron C., Salas-Alanis J.C., Dayal J.H., Cepeda-Valdes R., Yan W., Wright S., Chen M., Fine J.-D., Hogg F.J. (2012). Fibroblast-derived dermal matrix drives development of aggressive cutaneous squamous cell carcinoma in patients with recessive dystrophic epidermolysis bullosa. Cancer Res..

[24] Gewirtz D.A. (2014). Autophagy and senescence in cancer therapy. J. Cell. Physiol..

[25] Bastow E.R., Last K., Golub S., Stow J.L., Stanley A.C., Fosang A.J. (2012). Evidence for lysosomal exocytosis and release of aggrecan-degrading hydrolases from hypertrophic chondrocytes, in vitro and in vivo. Biol. Open.

[26] Zhang Y., Han Q., You S., Cao Y., Zhang X., Liu H., Hu L., Liu C.-F. (2016). Rapamycin promotes the autophagic degradation of oxidized low-density lipoprotein in human umbilical vein endothelial cells. J. Vasc. Res..

[27] Khundadze M., Ribaudo F., Hussain A., Stahlberg H., Brocke-Ahmadinejad N., Franzka P., Varga R.-E., Zarkovic M., Pungsrinont T., Kokal M. (2021). Mouse models for hereditary spastic paraplegia uncover a role of PI4K2A in autophagic lysosome reformation. Autophagy.

[28] Malchiodi Z.X. (2024). Natural Killer Cells in the Pancreatic Ductal Adenocarcinoma Tumor Microenvironment. Ph.D. Thesis.

[29] Oliveira A.C.S., Rezende L., Gorshkov V., Melo-Braga M.N., Verano-Braga T., Fernandes-Braga W., Guadalupe J.L.d.M., de Menezes G.B., Kjeldsen F., de Andrade H.M. (2022). Biological and molecular effects of Trypanosoma cruzi residence in a LAMP-deficient intracellular environment. Front. Cell. Infect. Microbiol..

[30] Zheng Y., Chen Z., Han Y., Han L., Zou X., Zhou B., Hu R., Hao J., Bai S., Xiao H. (2020). Immune suppressive landscape in the human esophageal squamous cell carcinoma microenvironment. Nat. Commun..

[31] Jayaraman P., Parikh F., Newton J.M., Hanoteau A., Rivas C., Krupar R., Rajapakshe K., Pathak R., Kanthaswamy K., MacLaren C. (2018). TGF-β1 programmed myeloid-derived suppressor cells (MDSC) acquire immune-stimulating and tumor killing activity capable of rejecting established tumors in combination with radiotherapy. Oncoimmunology.

[32] Zheng G.X., Terry J.M., Belgrader P., Ryvkin P., Bent Z.W., Wilson R., Ziraldo S.B., Wheeler T.D., McDermott G.P., Zhu J. (2017). Massively parallel digital transcriptional profiling of single cells. Nat. Commun..

[33] Wolf F.A., Angerer P., Theis F.J. (2018). SCANPY: Large-scale single-cell gene expression data analysis. Genome Biol..

[34] Bartha Á., Győrffy B. (2021). TNMplot.com: A Web Tool for the Comparison of Gene Expression in Normal, Tumor and Metastatic Tissues. Int. J. Mol. Sci..

[35] Tang Z., Kang B., Li C., Chen T., Zhang Z. (2019). GEPIA2: An enhanced web server for large-scale expression profiling and interactive analysis. Nucleic Acids Res..

[36] Li T., Fu J., Zeng Z., Cohen D., Li J., Chen Q., Li B., Liu X.S. (2020). TIMER2.0 for analysis of tumor-infiltrating immune cells. Nucleic Acids Res..

[37] Okato A., Goto Y., Kurozumi A., Kato M., Kojima S., Matsushita R., Yonemori M., Miyamoto K., Ichikawa T., Seki N. (2016). Direct regulation of LAMP1 by tumor-suppressive microRNA-320a in prostate cancer. Int. J. Oncol..

[38] Subramanian A., Tamayo P., Mootha V.K., Mukherjee S., Ebert B.L., Gillette M.A., Paulovich A., Pomeroy S.L., Golub T.R., Lander E.S. (2005). Gene set enrichment analysis: A knowledge-based approach for interpreting genome-wide expression profiles. Proc. Natl. Acad. Sci. USA.

[39] Shi S.R., Shi Y., Taylor C.R. (2011). Antigen retrieval immunohistochemistry: Review and future prospects in research and diagnosis over two decades. J. Histochem. Cytochem..

[40] Bankhead P., Loughrey M.B., Fernández J.A., Dombrowski Y., McArt D.G., Dunne P.D., McQuaid S., Gray R.T., Murray L.J., Coleman H.G. (2017). QuPath: Open source software for digital pathology image analysis. Sci. Rep..

[41] Zeglis B.M., Lewis J.S. (2015). The bioconjugation and radiosynthesis of 89Zr-DFO-labeled antibodies. J. Vis. Exp. JoVE.

[42] Vosjan M.J., Perk L.R., Visser G.W., Budde M., Jurek P., Kiefer G.E., Van Dongen G.A. (2010). Conjugation and radiolabeling of monoclonal antibodies with zirconium-89 for PET imaging using the bifunctional chelate p-isothiocyanatobenzyl-desferrioxamine. Nat. Protoc..

[43] Turker N.S., Heidari P., Kucherlapati R., Kucherlapati M., Mahmood U. (2014). An EGFR targeted PET imaging probe for the detection of colonic adenocarcinomas in the setting of colitis. Theranostics.

