# Supplementary material for: LAMP1 as a Target for PET Imaging in Adenocarcinoma Xenograft Models
Source: Pharmaceuticals (Basel). 2025 Jul 27;18(8):1122. doi: 10.3390/ph18081122 (PMC12388936; doi:10.3390/ph18081122)
Supplement: Supplementary file 1 [file pharmaceuticals-18-01122-s001.zip › pharmaceuticals-3722954-supplementary.pdf]

Supplementary figures

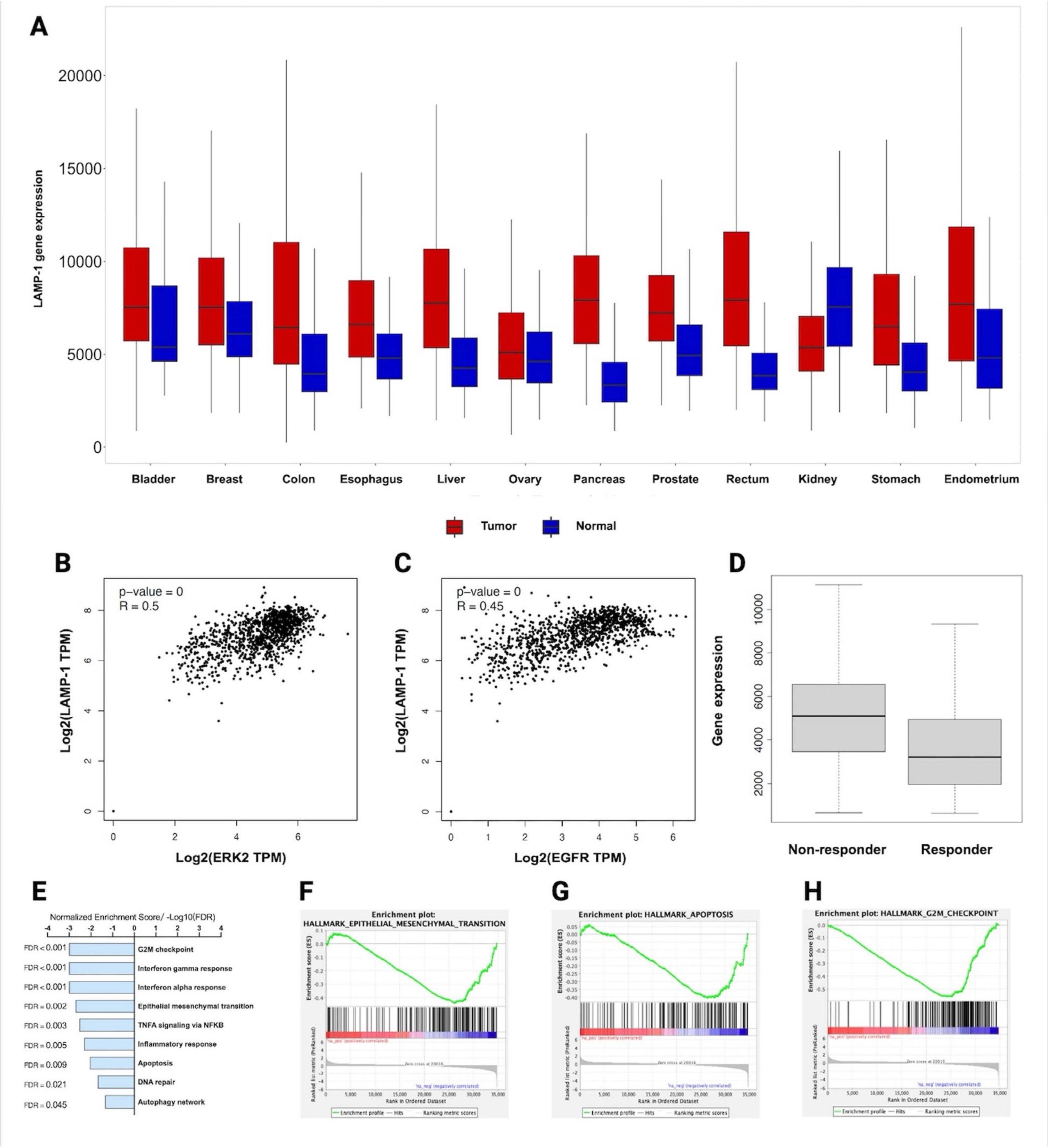

Supplementary Figure S1. (A) Pan-cancer LAMP-1 RNA expression in tumors (blue) and normal tissue (red). (**B**) Positive significant correlation between LAMP-1 and ERK-2 expression. (**C**) Positive significant correlation between LAMP-1 and EGFR expression. (**D**) LAMP-1 expression was higher in pan-cancer doxorubicin non-responders compared to responders. (**E)** The top significant down-regulated signatures in Hallmark gene sets for RNASeq analysis. The blue highlighted down-regulated signatures. GSEA analysis on signatures of (**F)** Epithelial mesenchymal transition. (**G**) Apoptosis and **(H)** Autophagy.

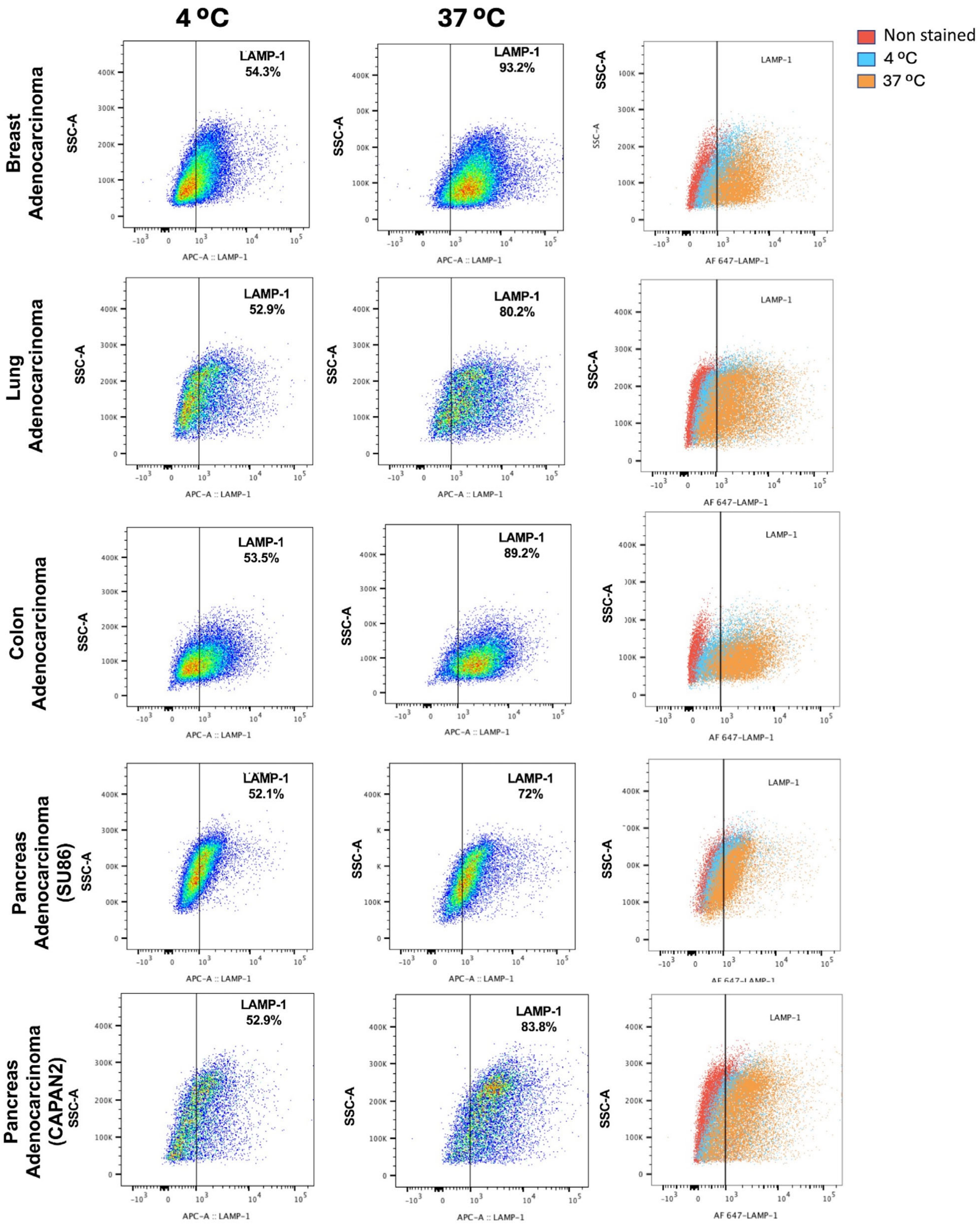

Supplementary Figure S2. Flow cytometry analysis of LAMP1 expression in cancer cell lines under different temperature conditions.

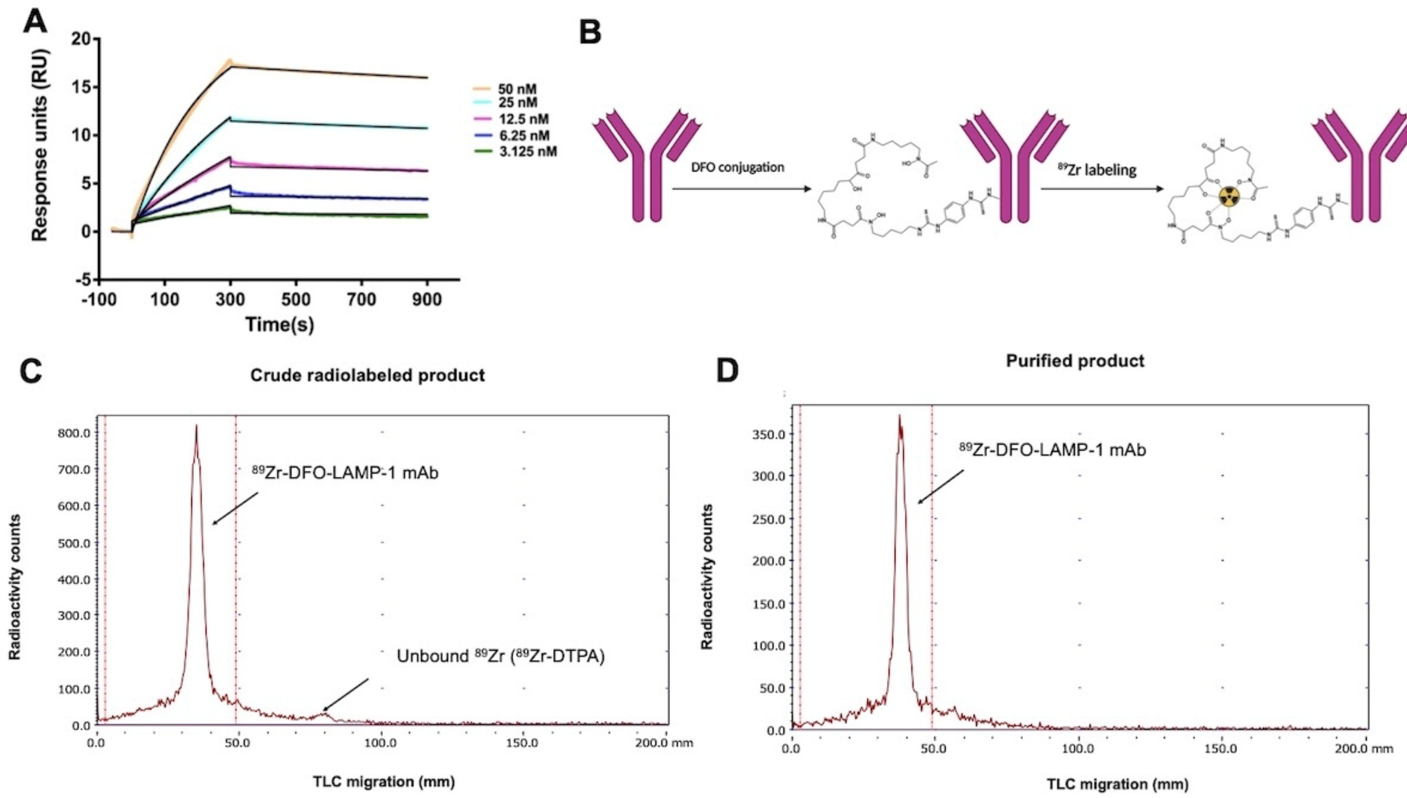

Supplementary Figure S3. (**A**) Binding affinity by SPR. Representative SPR sensorgrams show the response over time (resonance units, RU) during the association and disassociation binding phases of immobilized anti-LAMP-1 IgG Ab to recombinant human LAMP-1 (1:1 fit is shown as black curves). The anti-LAMP-1 IgG binds LAMP-1 at an affinity of KD = 1.78 ± 0.84 nM. (**B)** Schematic representation of DFO conjugation and ^{89}Zr labeling. (**C**) Representative radio-TLC chromatograms on ^{89}Zr-DFO-LAMP-1 of crude radioimmunoconjugate. (**D**) After purification, the unbound ^{89}Zr was removed from the final product.

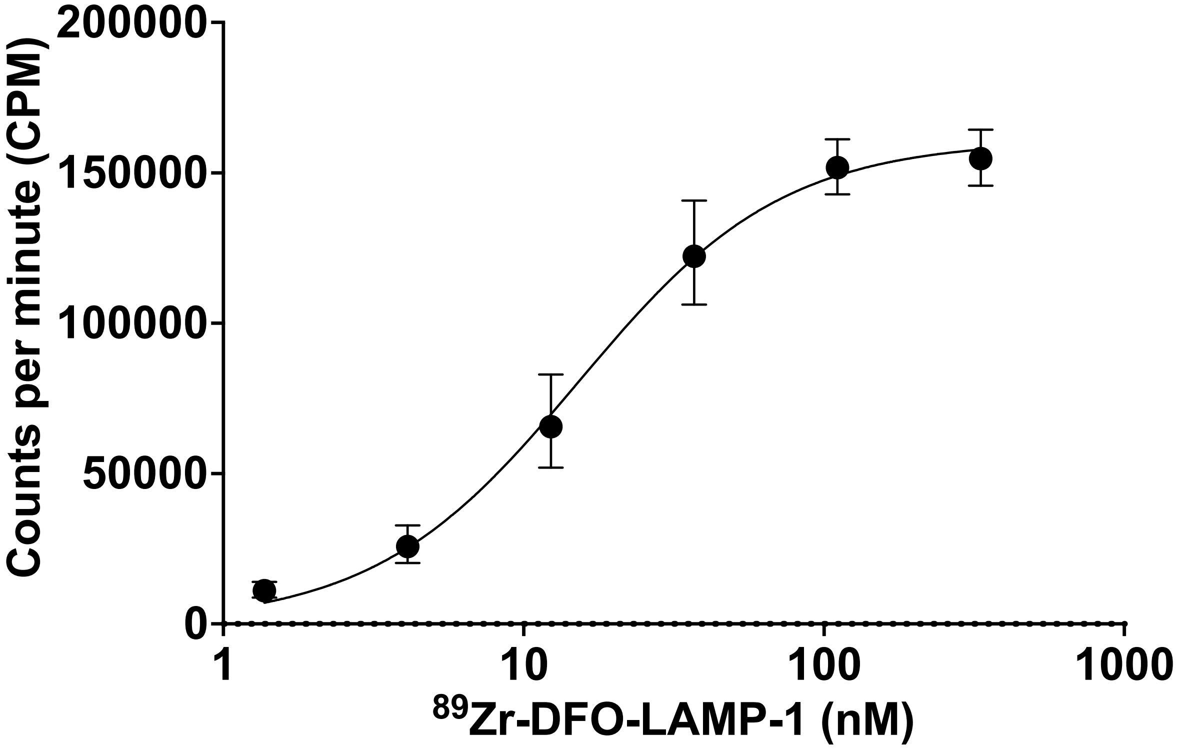

Supplementary Figure S4. Dose–response curve for specific ligand binding in the saturation cell-binding assay. Mean counts per minute (CPM) at each ligand concentration were fitted to a one-site binding isotherm, showing a binding affinity (Kd) of 15.2 nM

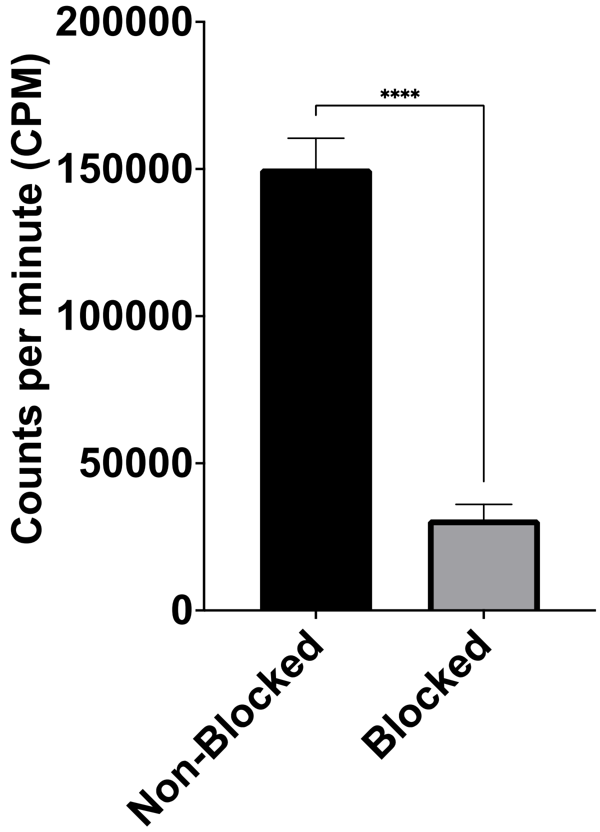

Supplementary Figure S5. Cell blocking assay shows that blocking with 10× molar excess cold anti-LAMP-1 mAb significantly reduces the cell uptake in DUBCA cells transduced with human LAMP-1 compared to non-blocked cells, indicating specificity for the target. ****, $p < 0.0001$

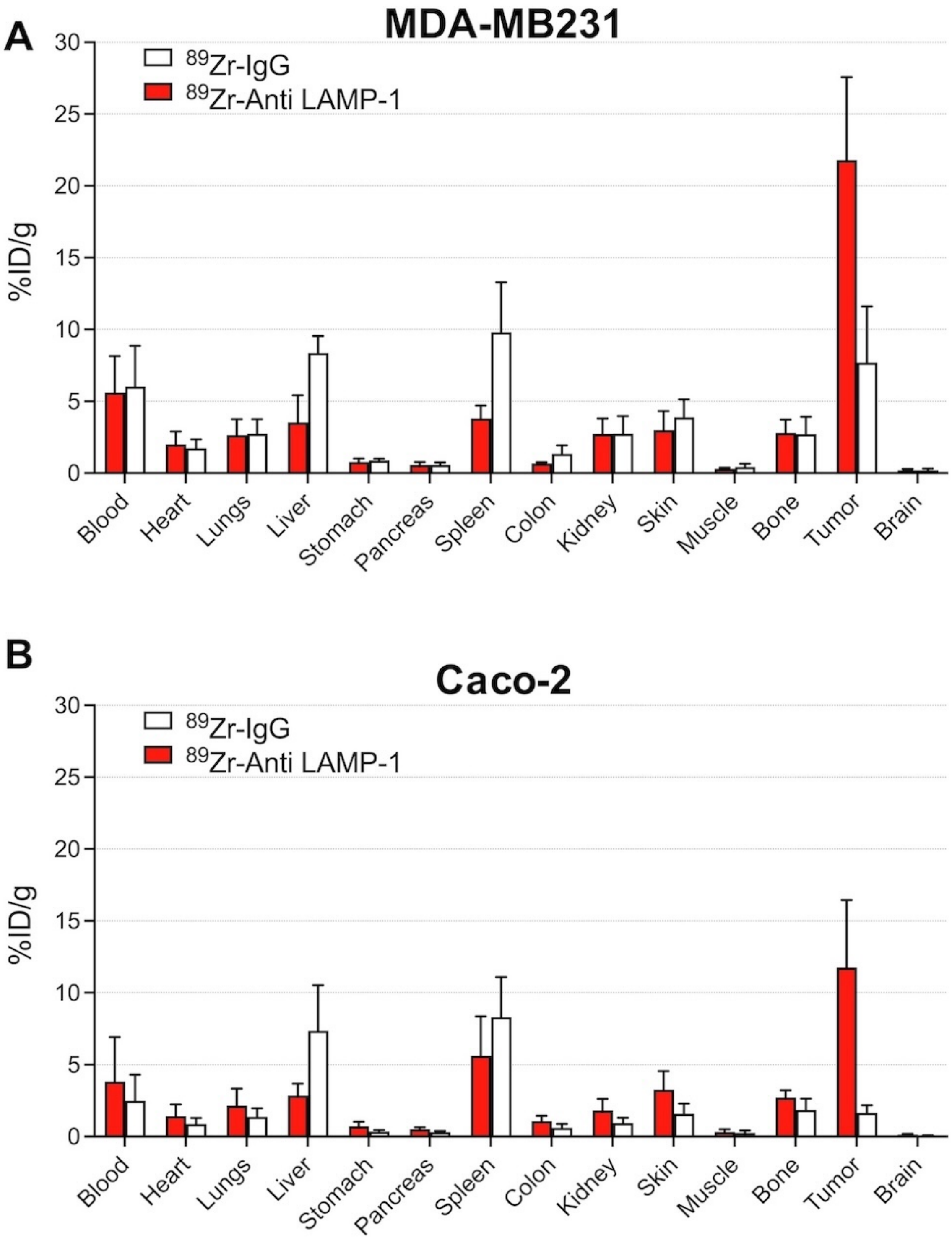

Supplementary Figure S6. Biodistribution of ^{89}Zr-DFO-LAMP1 (red) and ^{89}Zr-DFO-IgG (white) in tumor and major organs in (**A**) MDA-MB-231 and (**B**) Caco2 demonstrated high tumor uptake and low normal organ retention of ^{89}Zr-DFO-LAMP1.
